# A Combined Experimental and Computational Study of Chrysanthemin as a Pigment for Dye-Sensitized Solar Cells

**DOI:** 10.3390/molecules26010225

**Published:** 2021-01-04

**Authors:** Atoumane Ndiaye, Alle Dioum, Corneliu I. Oprea, Anca Dumbrava, Jeanina Lungu, Adrian Georgescu, Florin Moscalu, Mihai A. Gîrţu, Aboubaker Chedikh Beye, Issakha Youm

**Affiliations:** 1 Department of Physics, Cheikh Anta Diop University of Dakar, 5005 Dakar-Fann, Senegal; atoumane2.ndiaye@ucad.edu.sn (A.N.); alle.dioum@ucad.edu.sn (A.D.); acbeye@gmail.com (A.C.B.); 2 Department of Physics, Ovidius University of Constanta, 900527 Constanta, Romania; cornel.oprea@univ-ovidius.ro (C.I.O.); jmatei@univ-ovidius.ro (J.L.); contact.adriangeorgescu@gmail.com (A.G.); flmoscalu@univ-ovidius.ro (F.M.); 3 Department of Chemistry and Chemical Engineering, Ovidius University of Constanta, 900527 Constanta, Romania

**Keywords:** dye-sensitized solar cells, density functional theory, cyanidin 3-glucoside, *Hibiscus sabdariffa* L.

## Abstract

The theoretical study of chrysanthemin (cyanidin 3-glucoside) as a pigment for TiO_2_-based dye-sensitized solar cells (DSSCs) was performed with the GAUSSSIAN 09 simulation. The electronic spectra of neutral and anionic chrysanthemin molecules were calculated by density functional theory with B3LYP functional and DGDZVP basis set. A better energy level alignment was found for partially deprotonated molecules of chrysanthemin, with the excited photoelectron having enough energy in order to be transferred to the conduction band of TiO_2_ semiconductor in DSSCs. In addition, we used the raw aqueous extracts of roselle (*Hibiscus sabdariffa*) calyces as the source of chrysanthemin and the extracts with various pH values were tested in DSSCs. The extracts and photosensitized semiconductor layers were characterized by UV-Vis spectroscopy, and DSSCs based on raw extracts were characterized by current density-voltage measurements.

## 1. Introduction

Dye-sensitized solar cells (DSSCs), the solar cells developed by B. O’Regan and M. Gratzel [1,2], generate electric energy by imitating natural photosynthesis. The DSSCs are basically composed of a photoelectrode (a wide bandgap semiconductor, such as titanium oxide, TiO_2_), sensitized with a dye, a redox electrolyte (e.g., I_3_^−^/I^−^), and a counter electrode [1,2,3]. Since their invention, a very large number of organic dyes and coordination complexes have been used as sensitizers for DSSCs, N719 being the most famous member of the ruthenium polypyridine complexes family [4]. Natural dyes, consisting of vegetal pigments like anthocyanins, betalains, chlorophylls, and carotenoids [5,6,7,8] from various flowers, fruits, leaves, and vegetables [9,10,11,12,13], have been tested as sensitizers for DSSCs. Although natural dyes give low solar cell efficiency, they have received significant attention due to their low cost, abundance, and environment friendliness.

The anthocyanins, which are probably the most interesting natural pigments for DSSCs, have some special properties which differentiate them from other flavonoids, in which class they belong. Thus, besides the usual chemical properties of polyphenols, the anthocyanins are weak diacids, hard and soft electrophiles through C2 and respective C4 atoms of the pyrylium ring, nucleophiles, likely to develop π-stacking interactions and to bind hard to metal ions. The flavylium cation, which usually represents the anthocyanins, is the single chemical species only in acidic aqueous solution (pH < 2), but with the increase of pH, the anthocyanins change to a mixture of colored and colorless forms in equilibrium through acid–base, water addition–elimination, and isomerization reactions [14,15].

Our study deals with the theoretical characterization of chrysanthemin (cyanidin 3-glucoside) as a potential pigment for DSSCs. A theoretical model based on density functional theory (DFT) calculations was used for the prediction of the proton affinity and pKa values of the anthocyanin, in order to be used as a sensitizer for TiO_2_-based DSSCs. In addition to the theoretical calculation, we chose the roselle (*Hibiscus sabdariffa* L.) calyx as a source of the chrysanthemin for DSSCs sensitization [16,17]. The roselle is primarily cultivated because of the consumption of its calyx; in the tissues of its calyx was identified cyanidin 3-glucoside, together with a few other anthocyanins, such as cyanidin 3-rutinoside, delphinidin 3-sambubioside, cyanidin 3-sambubioside, and delphinidin 3-glucoside. Namely, delphinidin 3-sambubioside and cyanidin 3-sambubioside were identified as predominant anthocyanins, and cyanidin 3-glucoside and delphinidin 3-glucoside as minor compounds in the aqueous extracts of dried calyces of *H. sabdariffa* [17,18]. The differences between the four anthocyanins are minor, consisting in the nature of saccharide bound to the same anthocyanidin (cyanidin 3-sambubioside compared to cyanidin 3-glucoside, delphinidin 3-sambubioside compared to delphinidin 3-glucoside), respective of an additional -OH group in the anthocyanidin structure (in delphinidin 3-glucoside compared to cyanidin 3-glucoside). Because only a weak dependence of electronic properties on the number of hydroxyl [19] and glycoside groups was demonstrated (i.e., a slight increase of the energy gap between the highest occupied molecular orbital (HOMO) and the lowest unoccupied molecular orbital (LUMO)), we can extrapolate the properties of cyanidin 3-glucoside to all four anthocyanins mainly found in the rosella calyces.

The pigments were extracted from rosella calyces in aqueous media and the raw extracts were used for the sensitization of TiO_2_ layers. The acidity of extracts was varied in order to demonstrate the influence of extracts pH on the DSSCs characteristics. The acidity influences the protons transfer between anthocyanins and solvent, thus the protonation/deprotonation of pigments, but the protons also interact with TiO_2_ and the semiconductor protonation is another factor which must be considered in explanation of experimental results.

## 2. Results and Discussion

### 2.1. Theoretical Characterization of Chrysanthemin

**Figure 1:**
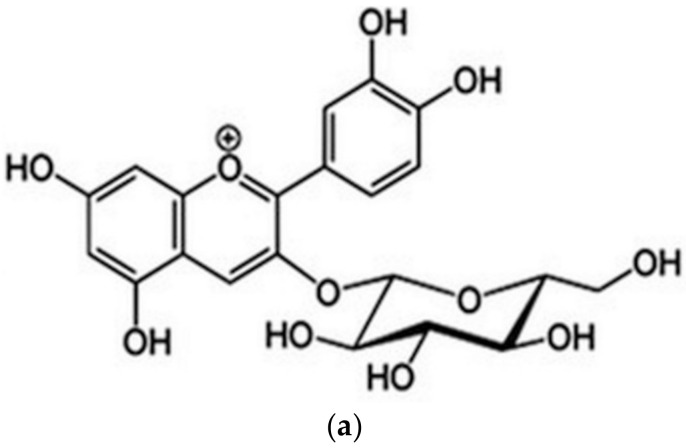

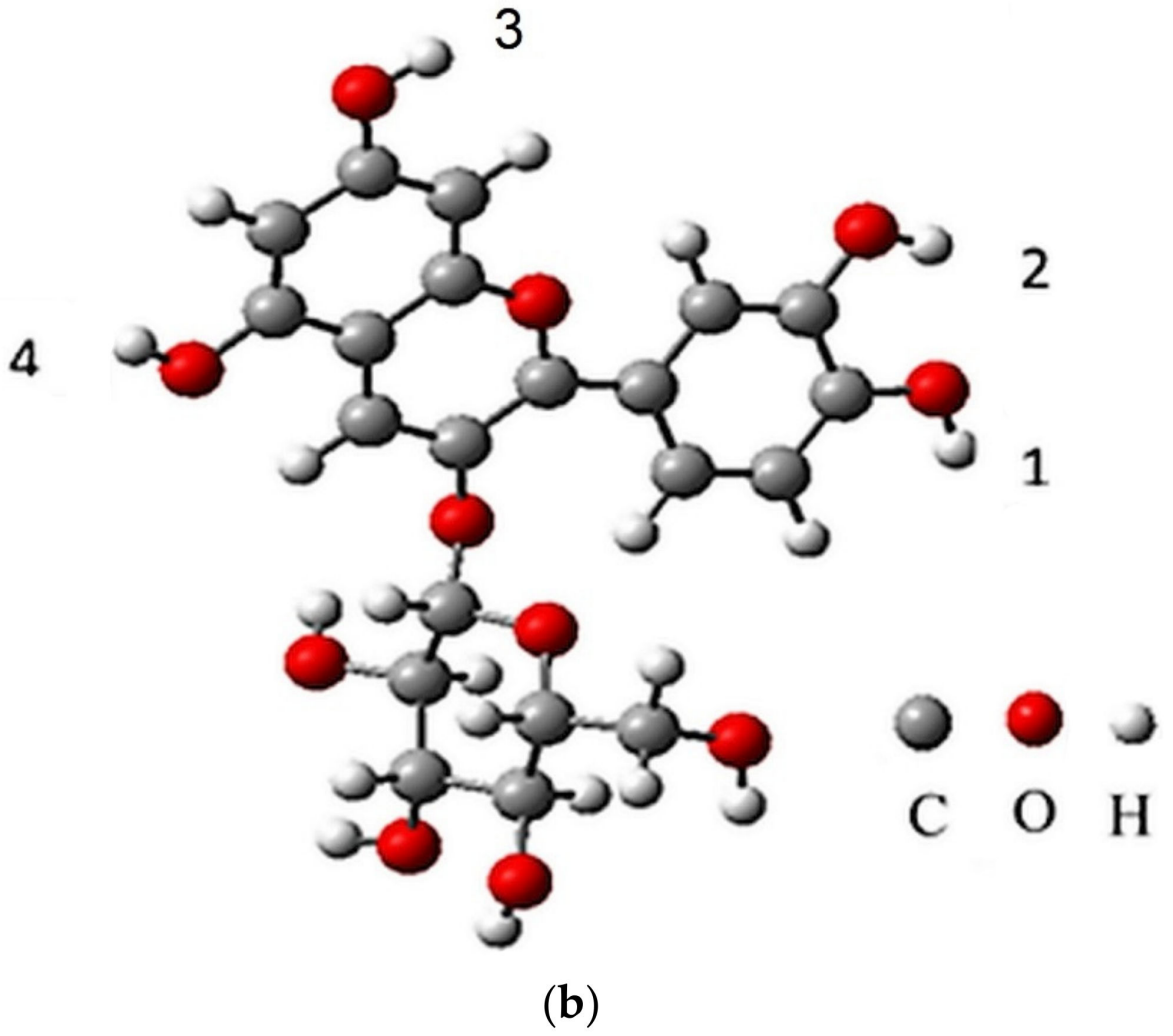
Cyanidin 3-glucoside structure (**a**) and labeling of the deprotonation sites (**b**).

The IUPAC name of cyanidin 3-*O*-glucoside (chrysanthemin) is (2*S*,5*S*)-2-[2-(3,4-dihydroxyphenyl)-5,7-dihydroxychromenylium-3-yl]oxy-6-(hydroxymethyl)oxane-3,4,5-triol, with the molecular formula C_21_H_21_O_11_^+^ (https://pubchem.ncbi.nlm.nih.gov/compound/Chrysanthemin), thus it is a mono positive molecular cation (Figure 1a). Based on the four hydroxyl groups (Figure 1b), chrysanthemin is a weak Brӧnsted acid, noted as AH_4_^+^, and the conjugate base resulted from chrysanthemin is AH_3_, followed by AH_2_^−^, AH^2−^, and A^3−^ in further deprotonation steps.

**Figure 2:**
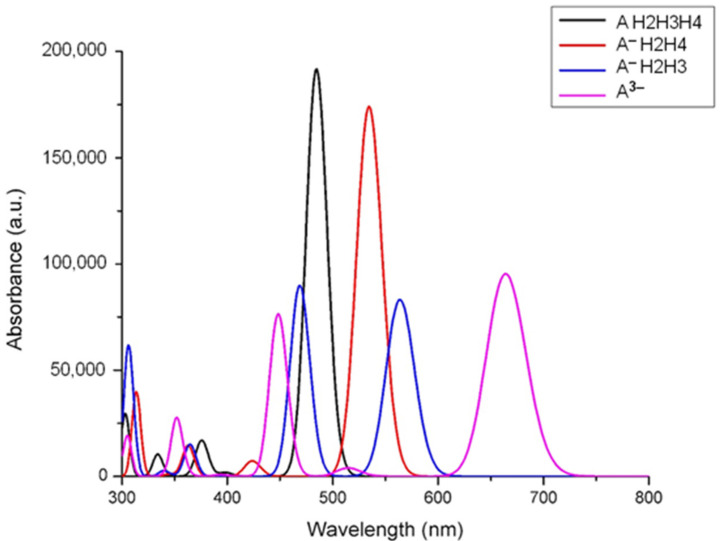
The calculated UV-Vis spectra of neutral and deprotonated species of cyanidin 3-glucoside.

The electronic spectra of mono deprotonated (denoted as A H2H3H4), double deprotonated species (two different mono-negative ions, noted A^−^), and total deprotonated cyanidin 3-glucoside (a tri-negative ion, noted A^3−^), calculated by time-dependent DFT for the lowest 20 singlet-singlet electronic transitions, are presented in Figure 2.

As can be seen in Figure 2, there is a large red shift in terms of the increase in deprotonation, and a dependence of the protonation site. The same dependence was observed for other anthocyanins, including in raw natural extracts [20]. Another observation is the decrease of intensity for the absorption bands as they shift to higher wavelengths, over 550 nm.

The value of the total dipole moment obtained by DFT calculation for the molecule increases and follows the trend of ground-state level of each deprotonated species. The excited state of the molecule becomes more polar compared to the ground state. The solvent stabilizes excited state more than ground state, so overall there is a decrease in the energy gap resulting the red shift upon the successive deprotonation, so an increasing polarity.

### 2.2. Chrysanthemin as Pigment for DSSCs

#### 2.2.1. Bonding to the Substrate

The ability of the dye molecules to bind on the semiconductor surface constitutes one of the relevant factors in the DSSCs performance. Our present work investigates the fulfillment of this condition by studying a natural dye with anchor groups such as hydroxyl, which is able to bind to atoms of the semiconductor, both by coordination and/or by hydrogen bonds [21]. Cyanidin 3-glucoside has four phenolic hydroxyl groups, and the computational calculation results indicate that hydroxyl proton is likely transferred to oxide during the dye adsorption on substrate.

**Figure 3:**
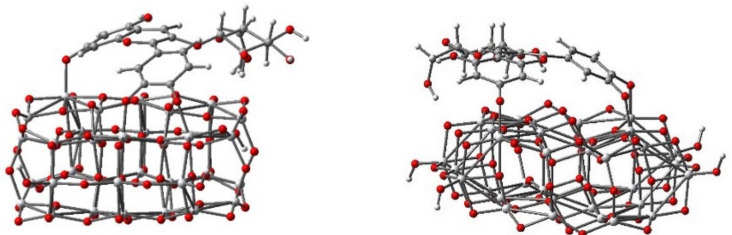
Adsorption modes of cyanidin 3-glucoside onto the titanium dioxide model cluster Ti_34_O_70_H_4_.

To identify both the most probable deprotonating site and the thermodynamically favorable hydroxyl group, which acts as an anchor, the proton affinity of dye was computed as energy difference between the deprotonated and protonated forms. Front and side views of the optimized geometrical structure of the adsorbed dye deprotonated at sites 1, 2, and 3 (labeled as in Figure 1b) are presented in Figure 3.

#### 2.2.2. Energy Level Alignment

**Figure 4:**
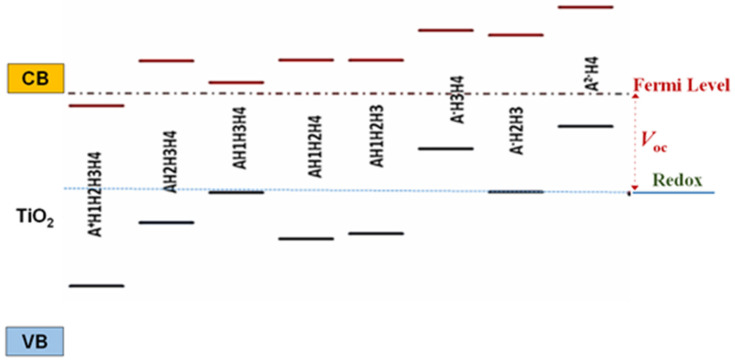
Diagram of energy level alignment of TiO_2_, various deprotonated species of cyanidin 3-glucoside, and the redox level of the electrolyte. Energy is relative to vacuum state.

The energy level alignment of the excited state of cyanidin 3-glucoside deprotonated molecules with the conduction band of semiconductor was analyzed, as it affects the charge transfer to the substrate, whereas the alignment of the ground state with the redox level of the electrolyte has an influence on the process of dye regeneration. The lowest unoccupied molecular orbital (LUMO) of dye should lie above the conduction band edge of TiO_2_, permitting the charge transfer from the dye to the oxide, followed by diffusion towards the contact. The highest occupied molecular orbital (HOMO) of the dye should be positioned below the redox level of the electrolyte allowing the electron transfer to the pigment for its regeneration. The energy level alignment in the case of partially deprotonated forms of the dye with respect to the band edges of TiO_2_ and the I^−^/I_3_^−^ electrolyte are displayed in Figure 4. Examining Figure 4 from left to right, we observe that the first structure (A^+^H1H2H3H4) has the excited state below the conduction band edge, failing to pass the energy alignment criterion. The sixth (A^−^H3H4) and the eight (A^2−^H4) structures have the ground state far above the redox level of the electrolyte and do not meet the alignment criterion either, whereas for the third (AH1H3H4) and the seventh (A^−^H2H3) models the dye regeneration might be possible but at low transfer rates. The criterion is fulfilled by AH2H3H4, AH1H2H4, and AH1H2H3, as it can be observed from the energy diagram in Figure 4.

The level of deprotonation may be correlated with the pH of the dye solutions, the outermost species, A^+^H1H2H3H4 and A^2−^H4, corresponding to acidic and basic solutions, respectively. Based on this correlation, we can interpret the results in Figure 4 by stating that the outermost pH levels fail the energy level alignment criterion. The good alignment predicts highest photovoltaic conversion efficiencies for the dyes solutions with intermediate pH levels.

S.A. Taya et al. [22] reported that the general decrease in conversion efficiency of DSSCs with the pH value variable of the dye solution can be attributed to the poor bonding between dye molecules and TiO_2_ film. As shown in Figure 3, the different deprotonation sites do influence the binding. More importantly; however, the diagram of energy level alignment (Figure 4) of the isolated molecule, with respect to the TiO_2_ and the electrolyte, indicates that for the outermost species, with lowest and highest pH levels, the electron transfer to the TiO_2_ and the electronic process of regeneration are not favorable and lead to the low efficiency of DSSCs.

#### 2.2.3. Electron Transfer

The electron transfer process is more probable when the electron density of excited states is localized close to the anchoring group bonded to the substrate. We investigated the delocalization of HOMO and LUMO of cyanidin 3-glucoside deprotonated in positions 1, 2, and 3, both in solution and adsorbed on the TiO_2_ surface modeled by the Ti_34_O_70_H_4_ cluster [23].

**Figure 5:**
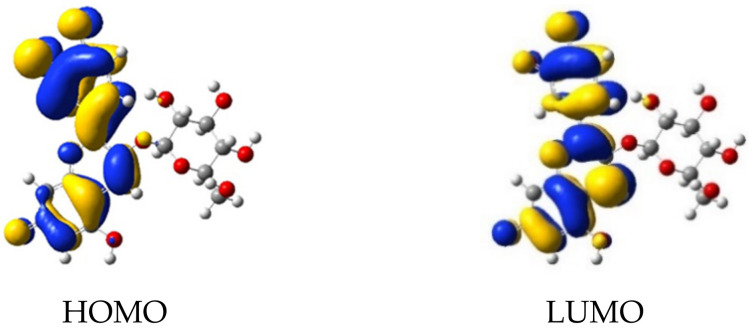
Molecular orbitals of cyanidin 3-glucoside deprotonated in sites 1, 2, and 3.

**Figure 6:**
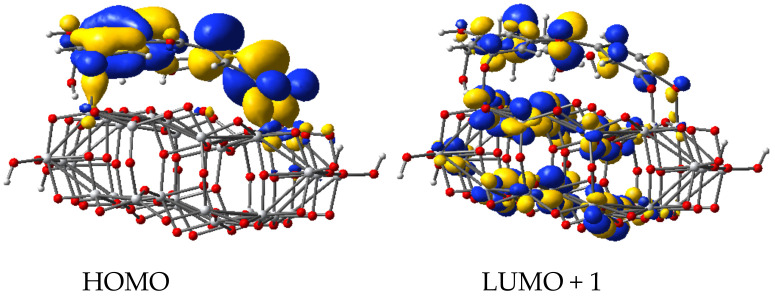
Molecular orbitals of deprotonated cyanidin 3-glucoside molecule adsorbed on Ti_34_O_70_H_4_ cluster.

Firstly, we determined the most stable geometries of dye/TiO_2_ systems, then we calculated the molecular orbitals and the lowest 100 singlet-singlet electronic transitions by including solvent effects via the conductor-like the polarizable continuum model (C-PCM), at the B3LYP/LANL2DZ level of time-dependent DFT (TD-DFT) (Table 1). We find *π*-type orbitals, with large electron density on the oxygen 1, 2, and 3 deprotonated sites for the HOMO, whereas for the π* character orbital, a large electron density is present on deprotonated anchor group, as depicted in Figure 5 and Figure 6.

**Figure 7:**
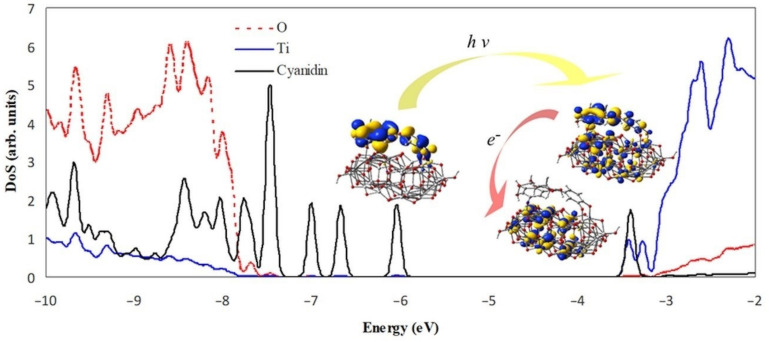
Projected density of states of cyanidin 3-glucoside molecule adsorbed on Ti_34_O_70_H_4_ cluster, together with key electronic states. The scale of the cyanidin contribution is double of the scale shown to the left. Energy levels were convoluted with Gaussian distributions of 0.1 eV full width at half maximum.

The electron transfer process is schematically displayed in Figure 7. The density of states projected on the adsorbed molecule and the constituent elements of the substrate reveals the mixed character of the photoelectron state. The diagram illustrates the energy level alignment, the position of the MOs involved in the excitation under visible light and in the electron transfer from the dye molecule to the semiconductor. The excited state (LUMO + 1) is just above the conduction band edge (LUMO) and has a large overlap with the LUMO. The key molecular orbitals are grouped to point out the optoelectronic processes, but they also present the anchoring, the charge delocalization, as well as the pathways for charge flow.

#### 2.2.4. pKa and Proton Affinity

The acid properties of the chrysanthemin were considered in the theoretical study of its application as pigment in DSSCs. We calculated the values of pKa and proton affinity (PA) as an indicator of Brӧnsted acid behavior. Thus, the value of pKa is usually used to show the strength of a Brӧnsted acid. A difficulty of the calculation of accurate pKa values can be the ambiguity of the deprotonation mechanism. The calculations were performed using isomolecules/ions in the gas phase and in the aqueous phase. We use water as solvent because of its strong hydrogen bond donor and acceptor, influencing on the acid/base properties.

**Figure 8:**
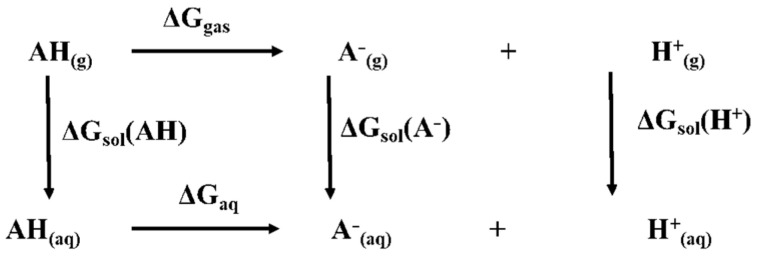
Scheme of the thermodynamic cycle.

The thermodynamic cycle for pKa calculation involves the determination of the gas phase deprotonation free energy change and the calculation of the hydration free energy for the molecule and the anion. Experimental values of the formation free energy (−628 kcal/mol) and of the hydration free energy change (−264.61 kcal/mol) are used for the proton [24]. Figure 8 shows the scheme of thermodynamic cycle used for the pKa calculation in Equation (1) [25].

(1) $$pKa=\frac{G_{\mathrm{gas}}\left(A^{-}\right)\;+\;G_{\mathrm{gas}}\left(H^{+}\right)\;-\;\Delta G_{\mathrm{gas}}\left(\mathrm{AH}\right)\;+\;\Delta G_{\mathrm{solvation}}\left(A^{-}\right)\;+\;\Delta G_{\mathrm{solvation}}\left(H^{+}\right)\;-\;\Delta G_{\mathrm{solvation}}\left(\mathrm{AH}\right)}{2.303RT}$$

where *G*_gas_(A^−^) and *G*_gas_(AH) are the gas-phase free energies (in kcal mol^−1^) of the anion and the molecule, respectively; Δ*G*_solvation_(A^−^) and Δ*G*_solvation_(AH) (in kcal mol^−1^) are the hydration energies of the anion and of the molecule, respectively.

In particular, the pKa values at 25 °C can be obtained using Equation (2):

(2) $$pKa=\frac{G_{\mathrm{gas}}\left(A^{-}\right)\;-\;\Delta G_{\mathrm{gas}}\left(\mathrm{AH}\right)\;+\;\Delta G_{\mathrm{solvation}}\left(A^{-}\right)\;-\;\Delta G_{\mathrm{solvation}}\left(\mathrm{AH}\right)\;-\;269.0}{1.3644}$$

The gas-phase and hydration free energies can be determined by vibrational frequencies calculations employing DFT with the B3LYP exchange correlation functional [26,27] and the DGDZVP basis set [28].

The accurate theoretical calculation values of proton affinities and pKa for cyanidin 3-glucoside in different deprotonating forms are presented in Table 2. The pKa_1_ experimental value determined in aqueous solution for deprotonation of cyanidin-3-*O*-β-glucoside in flavylium cation form is 5.88, [29]. The experimental value may correspond to the loss of H4 from AH2H3H4 (Table 2).

### 2.3. DSSCs Based on the Roselle Calyx Extracts

We used roselle calyces as a source of chrysanthemin in the manufacturing of DSSCs.

#### 2.3.1. Extracts Characterization

The pigments from the roselle calyces were easy and quickly extracted into water, without adding any acid (as it is usually used for other vegetal materials [30]). The raw extract is acid, pH = 3. The acid character of the aqueous extract, and so the slightly sour taste, is one of the reasons for its widespread use in the beverage industry.

**Figure 9:**
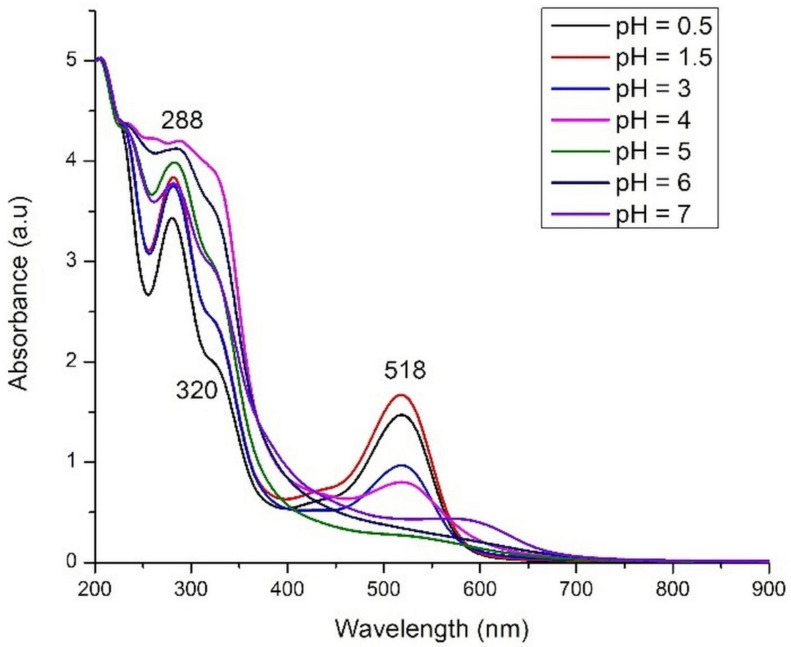
Experimental UV-Vis spectra of roselle calyces extracts at different pH values.

The pH value was slowly decreased to 2, but for pH = 0.5 the decrease was difficult. pH was increased by adding a solution of 1 M NaOH in the initial extract; thereby a dark blue-green coloration appears to disappear quickly, and the colorless anthocyanins were obtained. The decrease of absorption bands intensity with pH increase was also demonstrated by calculations (Figure 2). Contrary to other anthocyanins extracts [20,31], we failed in stabilizing the blue form of anthocyanins. In weak acidic medium (pH 5 and 6) an almost colorless solution was obtained. At pH values above 7, a brown precipitate, probably of anthocyanins degradation products [32], was obtained. The absorption spectra of the extracts are plotted in Figure 9.

In the visible range only an absorption band, centered at around 518 nm, can be identified in all spectra of acidic extracts (pH = 0.5–4). Very weak shoulders can be seen in 400–450 range for extracts with lowest pH values (0.5–3). In the UV-Vis spectra of *Hibiscus sabdariffa* ethanolic extract, two maxima of absorption, at 545 and 664 nm, were identified by Taya et al. [22]. This means that the aqueous extract is purer enough, containing only one type of pigments (i.e., anthocyanins), but also that the visible spectrum, so the color of the extract, is solvent-dependent.

The red color of the extract is intensifying at lower pH values and stronger absorption bands were registered in comparison with the spectrum of initial extract (pH = 3). The maximum absorption around 518 nm, which was observed for the roselle extracts, is in a good agreement with theoretical results from Figure 2. By comparison with theoretical spectra, the most intense band (i.e., pH = 1.5) can be assigned to the mono deprotonated molecule. The position of main absorption band for all extracts in 0.5–4 pH range is intermediate between mono deprotonated and double deprotonated species (around 480–560 nm) suggesting an overlap of bands and thus the existence of a mixture of these molecules. In the strong acidic medium, the hydrolysis of glycosidic bonds in anthocyanins and the existence of anthocyanidins (aglycone form) is very probable. In basic medium, the disappearance of the band assigned to anthocyanins is obvious. A very weak shoulder can be identified in the domain of 500–600 nm for the extract with pH = 7.

#### 2.3.2. Photoanode Characterization

**Figure 10:**
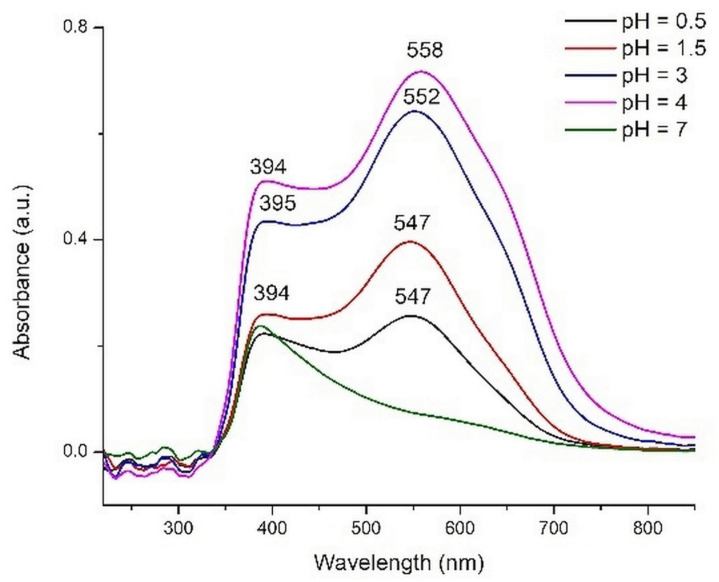
The UV-Vis spectra of the pigments adsorbed onto sensitized TiO_2_ layers.

We chose to use the extracts with pH values of 0.5, 1.5, 3, 4, and 7 to test as pigments for DSSCs. The electronic spectra of adsorbed pigments, obtained by subtracting the UV-Vis spectrum of TiO_2_ layer from the sensitized layers spectra, are shown in Figure 10.

As a general observation, the band characteristic to anthocyanins is shifted to higher wavelengths for all extracts. This shifting has as effect a change of the plates color compared to the corresponding extract and is due to the interaction with TiO_2_. The pigments molecules can be coordinated to Ti(IV) or the color can be changed due to the basic character of the oxide.

The most intense color was obtained for the plate sensitized with the extract having pH = 4. The violet color of the semiconductor layer is due to the shifting of absorption band to higher wavenumbers (558 nm) in comparison with red layers (547 nm) obtained in acidic media. Other components, probably yellow flavonoids from the rosella calyces, to which can be assigned the bands at 394 nm, are also adsorbed onto the TiO_2_ surface. The ratio between the intensities of these two bands (394/547 nm or 558 nm) decreases with the pH increase in acidic medium. The plates immersed in basic extracts have a totally different aspect, their color being yellowish.

In Section 2.2.2 we analyzed the energy level alignment as one of the criteria that need to be met by a dye to be a candidate for TiO_2_ sensitizer. Another criterion refers to the matching of the absorption spectrum of the dye with the solar irradiance spectrum. The UV-Vis spectra of the dyes displayed in Figure 10 clearly indicate that the dye solutions with the intermediate pH levels (3 and especially 4) have the strongest absorption in the visible range, where the solar irradiance is at its peak. In contrast, the outermost pH levels (0.5 and especially 7) perform poorly with respect to the spectral matching criterion.

**Figure 11:**
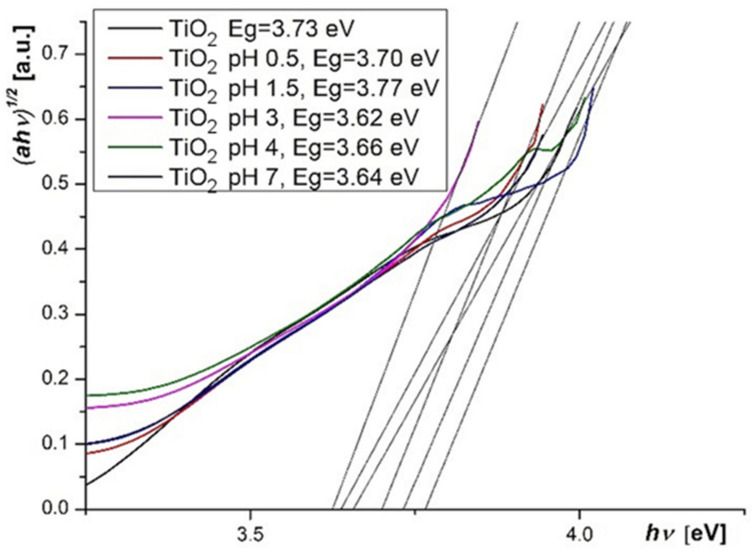
Tauc plots for TiO_2_ layers.

The bandgap energy of TiO_2_ deposited onto electrodes was calculated from the UV-Vis spectrum, by using the Tauc equation [33], and a value of 3.73 eV was determined. The variation of energy bandgap with the dye solution pH in sensitized photoelectrodes was determined also with the Tauc equation (Figure 11).

A relative irregular variation of E_g_ can be observed, but the values remain very close in the 3.62–3.77 eV range. The variation can be due to the TiO_2_ protonation, the effect of protonation onto the conduction band edge being previously noted [34]. The oxygen vacancies, obtained by the annealing or by other subsequent treatments, also influence the E_g_ value and results in the increasing of the semiconductor electrical conductivity [35]. The highest value of E_g_ was obtained for TiO_2_ layer sensitized at pH = 1.5, and it can be correlated with the highest values for fill factor (FF) and efficiency of DSSCs.

#### 2.3.3. Photoelectrochemical measurement

**Figure 12:**
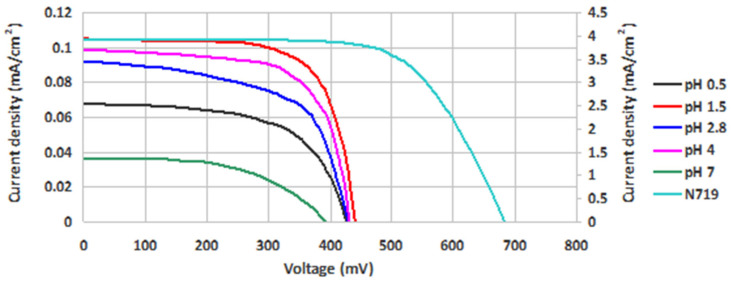
*I*-*V* curves of DSSCs prepared with the roselle calyx extracts with different pH values, in comparison with a reference DSSC.

The *I*-*V* measurements of the DSSCs sensitized with the extracts of *Hibiscus sabdariffa* L. are illustrated in Figure 12. The values for the fill factor (FF), the shunt resistance (*R*_sh_), the series resistance (*R*_s_), and the photovoltaic conversion efficiency (*η*) are present in Table 3. Maximum power point can be read from the *I*-*V* curves.

The difference of the acidity in 1.5–4 pH range is not high (acidic medium), and the results revealed small differences in FF and efficiency values. The higher values for both FF and efficiency were obtained at pH = 1.5, probably due to the existence of dyes as partially deprotonated and to the interaction between protons generated by HCl and TiO_2_ surface. It was previously demonstrated that surface protonation of TiO_2_ retarded charge recombination and slowed down the electron diffusion as well due to the electrostatic interaction between electrons and protons [34]. A lower concentration of H^+^ in the pigment solution favors the hydrogen bonds between HO- groups and oxygen from TiO_2_ surface, and also a coordination of anthocyanins to Ti(IV) atoms, which can be evidenced by the slight color change. It is possible that the influence of H^+^ concentration was exercised not so much on the pigment as on the semiconductor protonation. By increasing the H^+^ concentration (pH = 0.5), an excess of protons had, as an effect, a decrease of DSSC efficiency.

Another issue which must be considered is the possible reaction of hydrolysis, with the formation of anthocyanidins. The hydrolysis is possible both in acidic and in basic media, and the resulted anthocyanidins have the advantage of a smaller molecule volume, thus a lower steric hindrance on the semiconductor surface.

The careful examination if Table 3 reveals that the dye solution with highest pH has led to devices with the lowest photovoltaic conversion efficiency. This result fits well with the results of electronic spectrum calculations shown in Figure 4 and of optical absorption experiments displayed in Figure 10. Indeed, the highest level of deprotonation, associated with the higher pH, indicated a failure to meet the energy level alignment criterion, whereas the poor matching with the solar spectrum causes a lower short-circuit current and, in the end, a smaller efficiency.

Moreover the shape of the *I*-*V* curve for pH = 7 (which deviates far from the perpendicular to the vertical and horizontal axes) indicates the presence of high losses, a fact confirmed by the lower fill factor in Table 3. Such losses are due to the high series resistance and low parallel resistance of the device, which may be due to non-ohmic contacts and leakage currents, respectively.

At the opposite limit, the lowest pH may be associated with lowest level of deprotonation. In this case, again, the criteria discussed in Section 2.2.2 and Section 2.3.2 are either not met or poorly satisfied, the resulting fill factor and overall efficiency being small.

The central pH values, corresponding to intermediate deprotonation levels in Figure 4, meet both criteria and lead to sizeable fill factors and efficiencies. The higher absorption of the dye with pH = 4 explains the higher short-circuit current and efficiency. Moreover, the solution with lower pH shows higher losses, particularly near the open-circuit limit, due to the higher series resistance.

Under these circumstances, somewhat puzzling is the performance of the pH = 1.5 dye solution, as it corresponds to an intermediate matching with the solar spectrum as discussed in Figure 10. The high fill factor and efficiency might be due to a better energy level alignment and higher rates of charge injection in the oxide and dye regeneration. However, based on the limited correspondence that we are able to draw between calculations and experiments, any further discussion would be speculative.

## 3. Materials and Methods

### 3.1. Materials

The chemicals of high purity, obtained from Sigma-Aldrich (Taufkirchen, Germany), (titanium (IV) oxide, TiO_2_, anatase, nanopowder, <25 nm particle; titanium (IV) chloride, TiCl_4_; ethyl cellulose; terpineol; potassium iodide, KI; anhydrous ethanol, CH_3_CH_2_OH; acetonitrile, CH_3_CN; ethylene glycol, (CH_2_OH)_2_; 37% hydrochloric acid, HCl), Loba Chemie (Mumbai, India) (sodium hydroxide, NaOH), Merck (Darmstadt, Germany) (iodine, I_2_), and Solaronix (Aubonne, Switzerland) (N719, Ruthenium 535-bisTBA, (Bu_4_N)_2_[Ru(4–carboxy-4’-carboxylato-2,2′-bipyridine)_2_(NCS)_2_]; Iodolyte TG-50; 5 mM H_2_[PtCl_6_]∙6H_2_O solution in 2-propanol), were used without further purification. Soda lime glass sheet (Solaronix) of 2.2 mm thickness, coated with a conductive layer of F-doped SnO_2_, a sheet resistance of 15 Ω/cm^2^, and an optical transmission greater than or equal to 80% (400–700 nm region) was used for electrodes.

### 3.2. Methods

#### 3.2.1. Computational Details

Optical and electronic properties of cyanidin 3-glucoside were analyses using DFT and time-dependent DFT (TD-DFT) [36]. Accurate results were obtained for both the ground and excited states upon the association of hybrid exchange correlation functionals and basis sets containing diffuse functions on the heavy atoms in the system. The ground state geometries of cyanidin 3-glucoside and its different types of deprotonation were optimized using the B3LYP exchange correlation functional [26] and the basis set DGDZVP [28]. Vibrational frequency calculations were performed to verify the stability of all optimized structures and to obtain the zero-point corrections of the energy and the Gibbs free energy. The molecular orbitals and electronic transitions were calculated in water with the polarizable continuum model (PCM) [37,38]. TD-DFT calculations [36] were performed with the same functional and basis set for the first 20 singlet-singlet excited states. All calculations were implemented with the GAUSSIAN 09 package [39].

#### 3.2.2. Vegetal Extracts

The extracts were obtained from dried calyces of *H. sabdariffa*, harvested in the flora of Senegal. The dried roselle calyces were ground in a mortar. On 5 g of the resulted small pieces and powder of roselle calyces was added 75 mL of distilled water. The mixture was magnetically stirred for 4 h. The solid vegetal material was removed by filtration. The filtrate was divided in 7 parts. One part of the extract was kept unmodified. The pH value for the other 6 parts of the extract was varied by adding a 1 M NaOH solution (pH = 4–7), 1 M HCl solution (pH = 1.5), and a respective 37% HCl solution (pH = 0.5).

#### 3.2.3. Dye-Sensitized Solar Cells Preparation

***Photoanodes.*** The conductive glass plates were pre-treated by immersing in a 40 mM TiCl_4_ solution (40 min, 70 °C). The TiO_2_ paste, obtained by the method described by Ito et al. [40], was deposited using the “doctor blade” technique onto photoanodes and the resulted layer was sintered. The TiO_2_ covered plates were immersed overnight in a 40 mM TiCl_4_ solution [29,41]. For reference cells, we used N719 pigment in anhydrous ethanol (60 mg in 100 mL) as sensitizer [42]. The photoanodes for DSSCs based on vegetal pigments were obtained by immersion of TiO_2_ plates in fresh vegetal extracts with different pH values. The plates were kept in an oven for 2 h at 60 °C. The non-adsorbed dye was washed away with solvent until the rinse liquid was colorless and then the plates were dried at 40 °C for 60 min. ***Platinum counter electrodes.*** Few drops of H_2_[PtCl_6_] solution were spread on the FTO glass and dried at 100 °C for 10 min, and then at 385 °C for 30 min [39]. ***Electrolyte.*** Iodolyte TG-50 (50 mM of tri-iodide in tetraglyme) was used as an electrolyte for the reference DSSC. The electrolyte obtained by dissolving of KI (0.5 M) and I_2_ (0.05 M) in a mixture of ethylene glycol and acetonitrile (4:1 *v*/*v*) was used for vegetal pigment-based DSSCs [43].

The DSSCs were assembled following the procedure described in literature [44].

#### 3.2.4. Characterization and Measurements

The UV-Vis absorption spectra of extracts were recorded in the range of 200–900 nm, on a Jasco V 550 spectrophotometer. The pH of extracts was measured by a pH-meter (Hanna Instruments pH210, Bucharest, Romania). The UV-Vis diffuse reflectance spectra of sensitized photoanodes were recorded in the range of 220–850 nm, on a Jasco V 550 spectrophotometer, with an integrating sphere, using MgO as the reference sample. The electro-optical parameters of the DSSCs, the short circuit current, *I_SC_*, the open circuit voltage, *V_OC_*, the fill factor, *FF*, and the photovoltaic conversion efficiency, *η*, were measured under AM 1.5 G standard conditions (1000 W/m^2^) at 25 °C, using a homemade class A small area solar simulator [45]. The cell surface was exposed to light through a circular slit of 10 mm diameter, resulting in a useful area of about 0.785 cm^2^. The current and voltage values were measured using two digital bench multimeters (Mastech MS8050, Morcin, Spain) and a decadic resistance box. All measurements were made at intervals of 45 s, allowing for each reading to stabilize [46].

## 4. Conclusions

We performed a combined experimental and computational study of cyanidin 3-glucoside, as a representative anthocyanin, in order to find the proper conditions to increase the performance of dye-sensitized solar cells prepared with natural dyes extracted from calyces of *Hibiscus sabdariffa* L. The method relies on the molecular modeling of various deprotonated species of the cyanidin 3-glucoside molecule and DFT calculations indicating better energy level alignment for partially deprotonated species. The outermost species showed failure to meet the energy level alignment criterion, either for the ground state or for the excited state of the dye. The analysis of HOMO and LUMO of the cyanidin 3-glucoside molecule deprotonated in positions 1, 2, 3, bound to the TiO_2_ surface, shows a large electron density on deprotonated anchor groups, which favors the electron transfer from the excited molecule to the semiconductor.

The experimental investigations referred mainly to absorption spectra, *I*–*V* characteristics, and the electrical parameters of the DSSCs. The other key criterion for TiO_2_ sensitizers, namely the matching of the optical absorption with the spectral solar irradiation, was found to be better met by the central pH levels. Again, the dye solutions with outermost pH had the lowest absorption in the visible range and led to the worst performing devices.

The combined experimental and computational studies allowed us to make some structure–property correlations and to offer some microscopic explanations of the experimental observations made on actual devices. In particular, the combined methods permit drawing a parallel between the pH of the actual solution and the degree of deprotonation of the molecular model. However, our results obtained, of DSSCs sensitized with raw rosella calyces, confirmed that the cells’ characteristics and performance depend also on other experimental factors, which are difficult to include in DFT calculations.

## Figures and Tables

**Table 1:** Excitation energy and the oscillator strength (*f*) for cyanidin 3-glucoside deprotonated in sites 1, 2, and 3 computed at the TDDFT/B3LYP/DGDZVP in water.

Species	Energy (eV)	Wavelength (nm)	Oscillator Strength (*f*)	MO Configuration (Coefficient)
Cyanidin 3-glucoside deprotonated in sites 1, 2, and 3	1.8184	681.83	*f* = 0.4237	H − 3 →L (−0.12961) H − 1 →L (−0.11120) H →L (0.69480) H ←L (−0.13067)

**Table 2:** Energy, proton affinity (PA), Gibbs free energy, and pKa values of the cyanidin 3-glucoside dye, in various forms of deprotonation. Calculations were performed in water solvent.

Original Species	Deprotonated Species	Zero-Point Corrected Energy (Hartree)	PA (kcal/mol)	Gibbs Free Energy (kcal/mol)	pKa
A^+^H1H2H3H4	AH2H3H4	−1639.529214	871.788	−1,028,859.26	−0.96
A^+^H1H2H3H4	AH1H2H4	−1639.524791	869.013	−1,028,857.07	0.68
A^+^H1H2H3H4	AH1H2H3	−1639.523299	868.077	−1,028,856.50	1.06
AH2H3H4	A^−^H2H3	−1639.086931	594.251	−1,028,581.86	6.15
AH2H3H4	A^−^H2H4	−1639.086807	594.174	−1,028,581.23	6.62
AH1H2H4	A^−^H1H2	−1639.076644	587.796	−1,028,575.27	9.38
A^+^H1H2H3H4	AH1H3H4	−1639.502941	855.302	−1,028,843.04	10.93
AH2H3H4	A^−^H3H4	−1639.061685	578.409	−1,028,566.40	17.49
A^−^H2H3	A^2−^H2	−1638.619739	301.084	−1,028,287.98	18.24
A^−^H2H3	A^2−^H3	−1638.608313	293.914	−1,028,281.17	23.23
A^−^H2H4	A^2−^H4	−1638.607290	293.272	−1,028,280.31	23.39
A^2−^H2	A^3−^	−1638.139932	-	−1,027,986.90	23.51

**Table 3:** Electrical characteristics of the solar cells prepared from the roselle calyces extracts, in comparison with a DSSC based on N719.

Sample	*Voc* (mV)	*J* (mA/cm^2^)	*Pmax* (μW)	*FF*	*Rsh* (Ω)	*Rs* (Ω)	*η* (%)
N719	683	4.992	1789.71	0.668	13,600	32.8	2.279
pH = 0.5	425	0.087	17.43	0.603	67,721	792.7	0.022
pH = 1.5	441	0.134	32.4	0.700	146,235	388.4	0.041
pH = 3	430	0.117	23.52	0.595	55,800	733.3	0.030
pH = 4	431	0.125	28.38	0.672	197,000	365.8	0.036
pH = 7	392	0.046	7.59	0.537	467,540	2602.5	0.010

## Data Availability

The data presented in this study are available on request from the corresponding author.

## Sample Availability:

Samples of the compounds are not available from the authors.
